# High Wall Shear Stress can Predict Wall Degradation in Ascending Aortic Aneurysms: An Integrated Biomechanics Study

**DOI:** 10.3389/fbioe.2021.750656

**Published:** 2021-10-18

**Authors:** M. Yousuf Salmasi, Selene Pirola, Sumesh Sasidharan, Serena M. Fisichella, Alberto Redaelli, Omar A. Jarral, Declan P. O’Regan, Aung Ye Oo, James E. Moore, Xiao Yun Xu, Thanos Athanasiou

**Affiliations:** ^1^ Department of Surgery and Cancer, Imperial College London, London, United Kingdom; ^2^ Department of Chemical Engineering, Imperial College London, London, United Kingdom; ^3^ Department of Bioengineering, Imperial College London, London, United Kingdom; ^4^ Politecnico di Milano, Milan, Italy; ^5^ MRC London Institute of Medical Sciences, Imperial College London, London, United Kingdom; ^6^ Barts Heart Centre, London, United Kingdom

**Keywords:** aortic surgery, aneurysm, computational fluid dynamics, CFD, magnetic resonance imaging, wall shear stress, computational pathology, vascular biomechanics

## Abstract

**Background:** Blood flow patterns can alter material properties of ascending thoracic aortic aneurysms (ATAA) via vascular wall remodeling. This study examines the relationship between wall shear stress (WSS) obtained from image-based computational modelling with tissue-derived mechanical and microstructural properties of the ATAA wall using segmental analysis.

**Methods:** Ten patients undergoing surgery for ATAA were recruited. Exclusions: bicuspid aortopathy, connective tissue disease. All patients had pre-operative 4-dimensional flow magnetic resonance imaging (4D-MRI), allowing for patient-specific computational fluid dynamics (CFD) analysis and anatomically precise WSS mapping of ATAA regions (6–12 segments per patient). ATAA samples were obtained from surgery and subjected to region-specific tensile and peel testing (matched to WSS segments). Computational pathology was used to characterize elastin/collagen abundance and smooth muscle cell (SMC) count.

**Results:** Elevated values of WSS were predictive of: reduced wall thickness [coef −0.0489, 95% CI (−0.0905, −0.00727), *p* = 0.022] and dissection energy function (longitudinal) [−15,0, 95% CI (−33.00, −2.98), *p* = 0.048]. High WSS values also predicted higher ultimate tensile strength [coef 0.136, 95% CI (0 0.001, 0.270), *p* = 0.048]. Additionally, elevated WSS also predicted a reduction in elastin levels [coef −0.276, 95% (CI −0.531, −0.020), *p* = 0.035] and lower SMC count ([oef −6.19, 95% CI (−11.41, −0.98), *p* = 0.021]. WSS was found to have no effect on collagen abundance or circumferential mechanical properties.

**Conclusions:** Our study suggests an association between elevated WSS values and aortic wall degradation in ATAA disease. Further studies might help identify threshold values to predict acute aortic events.

## Introduction

Ascending thoracic aortic aneurysm (ATAA) is a permanent and irreversible dilatation of the thoracic aorta. Many patients remain asymptomatic until acute presentation with rupture or dissection (AD), which is associated with a 50% early mortality rate (Howard et al., 2013). Death from aortic aneurysm-related emergencies occurs at a rate of 2.4/100,000, and is one of the most common causes of death amongst conditions requiring emergency surgery in high-income countries (Stewart et al., 2014). Survivors of acute events often need repeat intervention and have high rates of stroke and renal failure. This has a huge impact on their quality-of-life, and carries significant societal burden (Tsai et al., 2006).

The evolution in our understanding of the disease from large observational studies has led to the reliance on ATAA diameter as the primary predictor of future AD. Intervention is recommended in patients who have a maximal aortic diameter ≥55 mm, with lower thresholds (≥50 mm) for those with bicuspid aortic valves (BAV) or connective tissue disease (Hiratzka et al., 2010; Erbel et al., 2014). Interventions in ATAA can be complex and not without risk, involving open surgery (particularly for ascending and arch aneurysms), endovascular stenting, or a combination of both. This drives the need for an accurate prognostic prediction prior to subjecting patients to treatment that involves moderate risk. However, isolated ATAA diameter measurements are inadequate predictors of acute aortic events. International registry data (>4,400 dissection patients) has highlighted that 40% of dissections occur below 50 mm (and up to 60% below 55 mm) (Pape et al., 2007). In this light, clinicians at present are unable to accurately predict the prognosis of enlarged thoracic aortas from routine imaging. Indeed, diameter alone fails to take into account local flow patterns, aortic wall stresses, mechanical properties and wall thickness. A patient specific approach is required.

Despite having the potential to predict disease progression, biomechanical assessment of the aorta remains experimental with limited translation into clinical practice. Biomechanically, AD occurs when haemodynamic forces exceed the aortic wall strength leading to intimal tear and false lumen propagation. It is possible that long-term exposure to certain blood flow patterns lead to changes in aortic wall structure (i.e., remodeling) that predispose wall degradation and a higher risk of AD. The identification of which flow patterns correlate with changes in aortic wall structure might lead to a more rational and useful patient-specific predictor (Geiger et al., 2012; Condemi et al., 2017; Piatti et al., 2017; Bollache et al., 2018).

The degradation of extracellular matrix (ECM) structures in ATAA is well reported. Methods to quantify degeneration center around computational histology techniques to characterize microstructural elements, such as elastin and collagen. Studies employing these methods have linked abnormal WSS patterns to disruption of the aortic microarchitecture (Guzzardi et al., 2015; Bollache et al., 2018). However, studies that use flow-to-tissue spatial registration (that incorporate the whole aneurysm) have been limited.

This study aims to explore the relationship between haemodynamic parameters, material properties and composition of the aortic wall in ATAA using whole aneurysm samples, with a view to assess the predictive ability of flow on aortic wall degeneration. We hypothesize elevated WSS in ATAAs is associated with accelerated degenerative disease.

## Materials and Methods

The study was ethically approved (17/NI/0160) by the Health Research Authority (HRA) in the United Kingdom and was sponsored by the Imperial College London Joint Research and Compliance Office, as defined under the sponsorship requirements of the Research Governance Framework (2005). The study was designed as a cohort study during the years 2018-2020, incorporating computational flow analysis, *in-vitro* vascular wall mechanical testing and microstructural quantification. Patients and public were involved in the design, conduct, reporting, and dissemination plans of our research via the London Aortic Mechanobiology Working Group.

### Study Population

A total of 10 patients undergoing proximal aortic surgery (either aortic root, ascending aorta, proximal arch replacements, or a combination of these) were recruited into this cohort study. The main exclusion criteria were: connective tissue disease (i.e. Marfans, Ehler-Danlos, Loeys-Dietz), bicuspid aortic valve and redo-aortic surgery. Patient characteristics were ascertained from clinic letters and pre-operative echocardiography. Recruited patients provided informed consent for participation in the study, which involved the additional MR imaging and provision of tissue sample from surgery.

### MRI Acquisition

Patients were scheduled to undergo cardiac gated magnetic resonance imaging (MRI) scanning at a time point prior to surgery (range 1–90 days) using a 3T MRI scanner (Siemens Healthcare, Erlangen, Germany). The addition of a time-resolved 3-dimensional sequence (4D-flow) visualized and measured temporal changes and flow-patterns throughout the whole volume of the thoracic aorta.

### Aneurysm Tissue Characterization

**FIGURE 1:**
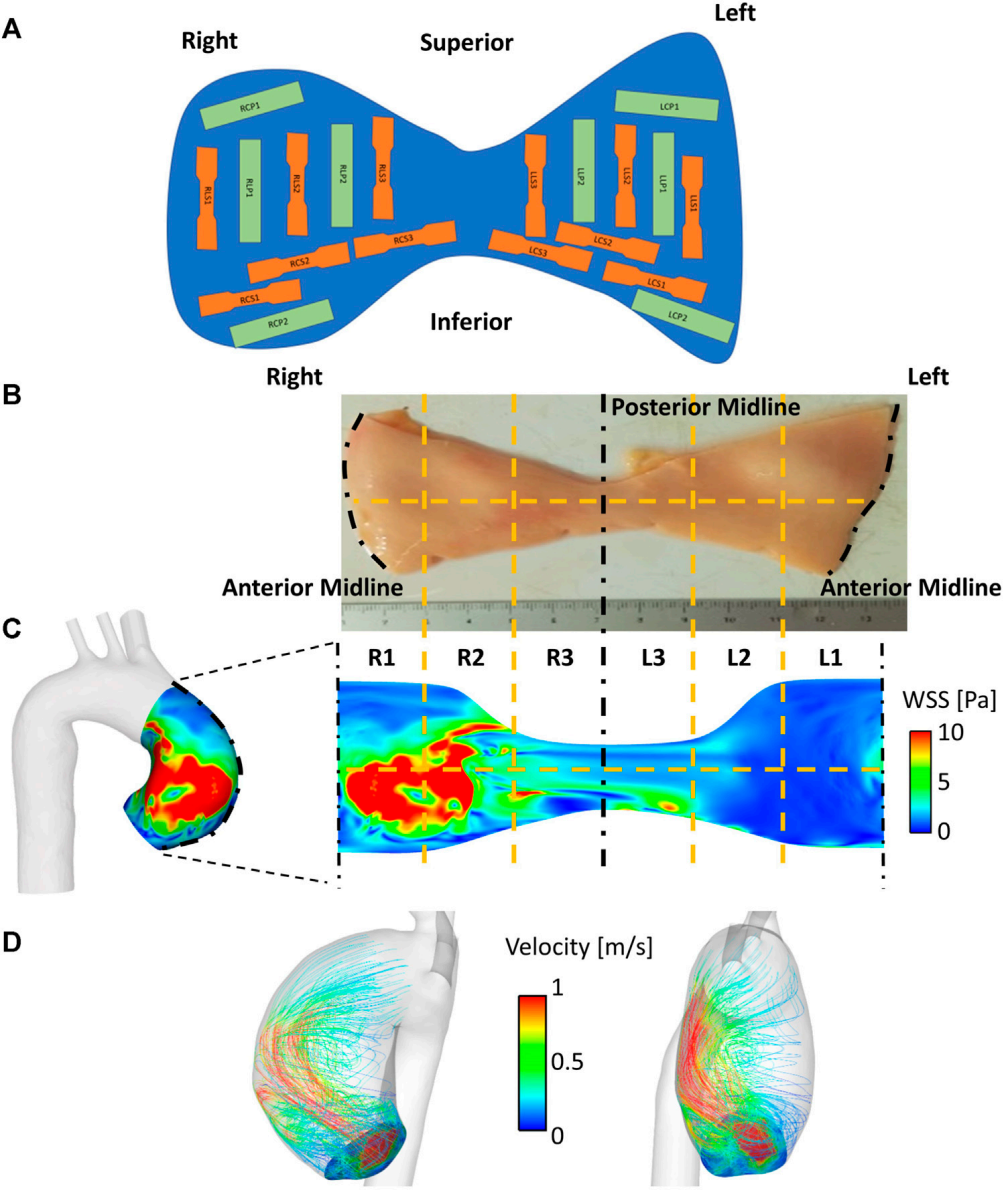
Matching flow to material properties. **(A)** Reference map used for collection of samples throughout the whole aortic specimen. Segments are classified using alphanumeric codes in which the first letter and number denote the position (R = right, L = left), the second letter denotes the sample orientation (C = circumferential, L = longitudinal), the third letter denotes the sample type (S = dogbone, P = rectangular) **(B–C)** WSS mapping on to aneurysmal wall surface, which is unraveled by division along the anterior. This is subdivided into six circumferential regions (R1-R3, L1-L3), with a horizontal dividing line (12 segments total). **(D)** Streamlines demonstrating haemodynamic flow derived from CFD within an anatomically (patient-specific) segmented aortic wall.

Patient-matched aneurysm specimens were obtained *en-bloc* and acquired immediately after surgical excision in the operating theatre. Rectangular or dog-bone shaped subsections aligned in either the longitudinal or circumferential direction were punched out at specific locations (Figure 1A) and assessed for tensile mechanical properties, peel strength and strain inhomogeneities (Olchanyi et al., 2020). In particular, for each patient, the aneurysm region was unfolded at the outer wall and subdivided into up to six (depending on the size of the collected sample) circumferential anatomical regions to the right (R1, R2, R3) and left (L1, L2, L3) of the midline (Figure 1B). These were further divided into upper and lower regions, giving 6 to 12 segments in total per patient (Figure 1B). These segments were matched to the 6–12 segmental regions undergoing computational WSS mapping (Section 2.4; Figure 1C), using the anterior midline (widest region of the aneurysm region in the vertical plane) as the common frame of reference. Whole-aneurysm tissue specimens underwent thickness mapping (∼0.01 µm resolution using a bench top device - Litematic VL-50-B Mitutoyo Ltd.).

Sharp stencils were used to create 20 mm × 5 mm dogbone samples (for uniaxial testing). The dogbone shaped tissue provided larger shoulders for easier gripping, whereas the narrower gauge section in the center provided a smaller cross-sectional area for a focused region of deformation and failure. The samples for peel testing required a different shape: the width of the peeling sample needed to be kept constant throughout the peeling arc. As such rectangular stencils were used (5 mm × 20 mm) to obtain eight further samples from both halves of the aneurysm specimen (both circumferential and longitudinal) –these would be used for peel testing.

Uniaxial tensile tests were performed on all dog-bone shaped subsections using a Test Resources R-Series Controller with a 44 N load cell. All tests were conducted in an environmental chamber containing phosphate buffered saline (PBS), maintained at 37°C. Tensile force-elongation data were used to provide estimations of region specific ultimate tensile strength (UTS) and maximum tangential stiffness (MTS) as described by previous groups (Vorp et al., 2003). Peel testing allowed estimation of peel force (F_peel_) and dissection energy function (DEF) for rectangular subsections.

### Computational Fluid Dynamics (CFD)

Image-based CFD modelling was used to evaluate pre-operative aneurysmal WSS distribution in a patient-specific and region-specific fashion. To this aim, the 3D thoracic aortic geometry, including the arch branches, of each patient was reconstructed from MRI data (bright blood). Reconstructed geometries (Figures 1C, D) started upstream of the aneurysm, in the aortic root between mid-sinus and STJ levels, and ended distally, in the descending thoracic aorta at the level of the pulmonary artery.

Patient-specific geometries were discretized using unstructured meshes (Supplementary Figure S1) with a tetrahedral core and 10 prismatic layers at the walls. Local mesh refinements were prescribed at the aneurysm wall and arch branches. Mesh refinement in the region of interest (i.e. aneurysm wall) was guided by flow features observed from 4D flow MRI data (i.e. a finer mesh was designed where higher velocity gradients were observed). Sensitivity analyses were conducted to ensure mesh-independent results. Final meshes consisted of 5.5–16.6 million elements (Pirola et al., 2019).

For each patient, 3D subject-specific velocity profiles of the aortic valve were incorporated at the computational model inlet from 4D flow MR images (Armour et al., 2021). This therefore took into account the patient-to-patient variation in the ascending aorta hemodynamics which arise due to differences in left ventricular outflow tract and aortic valve anatomy (Figure 1D). Model outlets, located distal to the aneurysm, consisted of the descending aorta outlet and the arch branches. 3-element Windkessel model was applied at all outlets; model parameters were tuned using patient-specific central mean aortic pressure and blood flow rates evaluated using 4D flow MRI data (Pirola et al., 2017; 2019). Briefly, the total resistance (R_T_) of the 3-element Windkessel model was calculated as R_T_ = P_m_/Q_m_ (Les et al., 2010). P_m_ is the mean central pressure of the patient, which was measured ∼30 min before the MR scan using a BP Plus device (BP Plus, Uscom, Australia). Q_m_ is the mean flow through the outlet. This was evaluated using 4D flow MR images. The proximal resistance was evaluated as R_1_ = ρc/A (Xiao et al., 2014, where *ρ* is the blood density, c is the pulse wave speed and A is the outlet cross-sectional area. The pulse wave speed was evaluated as c = 13.3/(2r)^0.3^ Reymond et al. (2009)), where r is the outlet radius. The distal resistance was evaluated as R_2_ = R_T_-R_1_ (LaDisa et al., 2011). Total vasculature compliance was calculated as C = τ/R_T_, where τ is the time-constant of the exponential diastolic pressure-fall (Xiao et al. (2014)). This was assumed equal to 1.79 and 1.92 s for normotensive and hypertensive subjects, respectively (Simon et al., 1979).

Simulations were run in ANSYS CFX (v 15.0). The aortic wall was assumed to be rigid with a no-slip condition. Blood was modelled as an incompressible Newtonian fluid, with a density of 1,060 kg m^−3^ and viscosity of 4·10^−3^ Pa s. High-resolution advection scheme and second-order Backward Euler scheme were employed for spatial and temporal discretization of the Navier-Stokes equations, respectively. A fixed timestep of 10^−3^ s was used, and the maximum RMS residual was set to 10^−5^ as a convergence criterion. The shear stress transport transitional (SST-Tran) model was used to account for the transitional nature of the aortic flow (Tan et al., 2009), with a 1% turbulence level prescribed as the inlet boundary condition. The patient-specific central diastolic pressure was used as the initial condition. Simulations were run for the number of cycles necessary to achieve periodicity. The period of the cardiac cycle of each patient was recorded during 4D flow MR acquisition and stored in the image header. Periodicity was considered as achieved when differences in pulse pressure and pressure maxima between two consecutive heartbeats were less than 3 and 1%, respectively, at each model outlet. Results from the last cycle only were analyzed. Before proceeding with result analysis, for each patient, predicted blood flow features were checked against 4D flow MR acquired features.

Computational result postprocessing workflow was designed to coincide with tissue processing for mechanical and histological testing: for each patient, the aneurysm region was unfolded at the outer wall and subdivided into segments corresponding to the aortic aneurysm tissue segments used to characterize the material properties (Figures 1B,C). Results were analyzed in ANSYS EnSight.

The primary derived measurement from CFD analysis was the magnitude of the wall shear stress (WSS) vector. For each anatomical region, several WSS-derived parameters were evaluated. These are summarized in Table 1. Briefly, WSS values are reported as maxima in time (WSS_max_, WSS_mean_) and time-averaged (TAWSS_max_, TAWSS_mean_, WSS^Tmean^_Max_). *Mean* and max subscripts refer to spatial mean and maxima, respectively. *Tmean* superscript refers to temporal mean. The spatial mean value was calculated over each segment (R1, R2, R3, L1, L2, L3, upper and lower).

**TABLE 1:** Calculated wall shear stress (WSS) parameters from computational fluid dynamics (CFD) analysis.

Short name	Description	Formula
WSS_max_	Maximum in time and space of the WSS magnitude	Time Max(Spatial Max(|WSS|)).
WSS_mean_	Maximum in time of the spatial mean of the WSS magnitude	$\text{TimeMax}\left(\frac{\sum {\left|\text{WSS}\right|}_{i}\cdot A_{i}}{\sum A_{i}}\right).$
		Where *i* refers to the surface mesh element at the aortic wall and *A*_*i*_ is the area of the surface mesh element. The mean value was calculated over each sub-segment
WSS^Tmean^_Max_	Mean in time of the spatial maximum of the WSS magnitude	$\frac{\sum {\left(\text{SpatialMax}\left(\left|\text{WSS}\right|\right)\right)}_{j}}{j}$
		Where *j* is the number of time points
TAWSS	Time average of the magnitude of the WSS	$\text{TAWSS}=\frac{1}{T}\int_{0}^{T}\left|\text{WSS}\right|\text{d}t$
TAWSS_max_	Spatial maximum of the TAWSS	SpatialMax(TAWSS)
TAWSS_mean_	Spatial mean of the TAWSS	$\frac{\sum {\text{TAWSS}}_{i}\cdot A_{i}}{\sum A_{i}}$

### Computational Pathology

**FIGURE 2:**
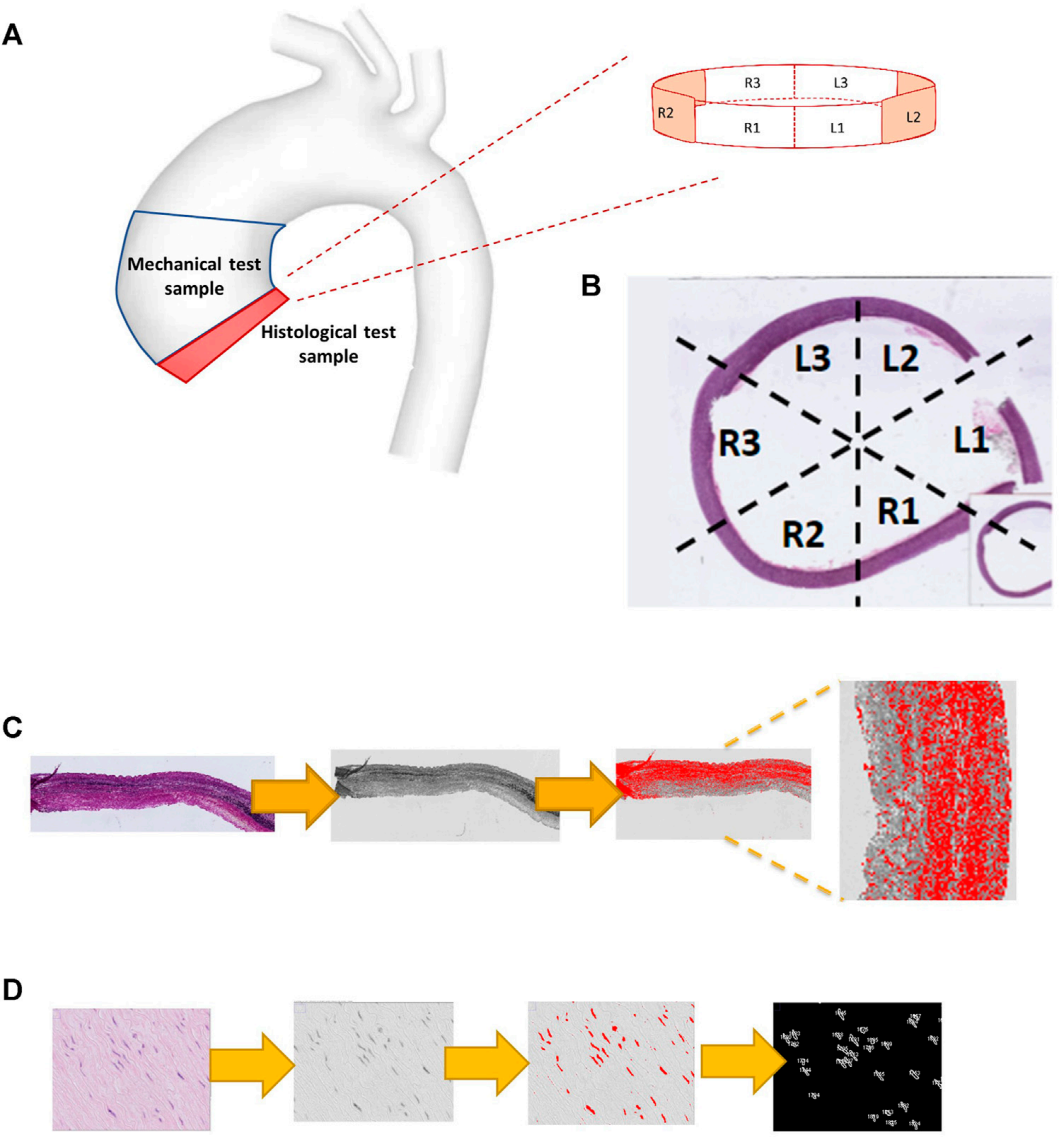
Computational pathology: regional histological analysis. **(A)** the origin of the histological specimen from the ATAA region. **(B)** Whole slide imaging demonstrating the division into six segmental regions. **(C)** Computational steps used to quantify elastin density (using EVG stained slide image), including RGB-stack image conversion, thresholding and measurement. Collagen quantification uses the same steps but for a PSR stained image. **(D)** displays the computational steps used to measure smooth muscle cell count (from an H&E-stained slide image) including particle analysis, using a size window of 20–100 μm.

Full circumferential rings of tissue were obtained from the inferior border of the ATAA specimen (Figure 2A). Whole slide imaging was performed using a high-resolution digital optical system (Hamamatsu TM) and uploaded onto the digital processing software QuPath (https://qupath.github.io). Cross-sectional images of each ATAA ring were divided into six circumferential regions (Figure 2B), matching the right-to-left segments used in the CFD and mechanical analyses (Figures 1B, C). Using a pre-defined workflow (Figure 2C), microstructural density calculation of medial structural proteins was conducted, namely of elastin (from slides stained with Elastin Van Gieson) and collagen (slides stained with picrosirius red). Using the H&E stained slides (Figure 2D), the thresholding function was used to highlight the stained cells of the medial layer. Setting the range of particle size from 20 to 100 µm the total cell count was extracted and divided by the total area to obtain the density.

### Statistical Analysis

Amongst the 10 recruited patients, a total of 371 ATAA anatomical samples had matched CFD, material properties and microstructural features, and were thus included in the analysis, being treated as separate data points. Of these samples, thickness was measured in 102 samples, 63 samples (29 longitudinal, 34 circumferential) were used for tensile testing, 63 samples (30 longitudinal, 33 circumferential) for peel testing, 143 for histological analyses (60, 35 and 48 for elastin, collagen and SMCs, respectively). All haemodynamic parameters were acquired as continuous variables, as were the histological and mechanical data. Where relevant, data were reported as means and standard deviations, being averaged across the whole patient cohort. All statistical analysis were conducted using STATA 13.0 (Stata Corp. College Station, TX, United States).

Univariable linear regression analysis was used to explore the effect of WSS on acquired material parameters (including mechanical and histological). These models tested the hypothesis of a flow-mediated degenerative process in the aortic wall giving rise to altered material properties. Results of regression analysis were reported as standardized beta-coefficients with 95% confidence intervals. The significance level was set at *α* = 0.05. Values of longitudinal F_peel_, longitudinal DEF, UTS and MTS were positively skewed; values of elastin abundance were negatively skewed. Therefore, logarithmic transformation with skewness correction was applied in STATA to these datasets.

Scatter plots and trendlines have been generated using Microsoft Excel, using log_10_ transformation for longitudinal DEF and UTS, and log_10_ (90-x) transformation for elastin abundance.

#### Multilevel Mixed Effects (Hierarchical) Linear Models

**FIGURE 3:**
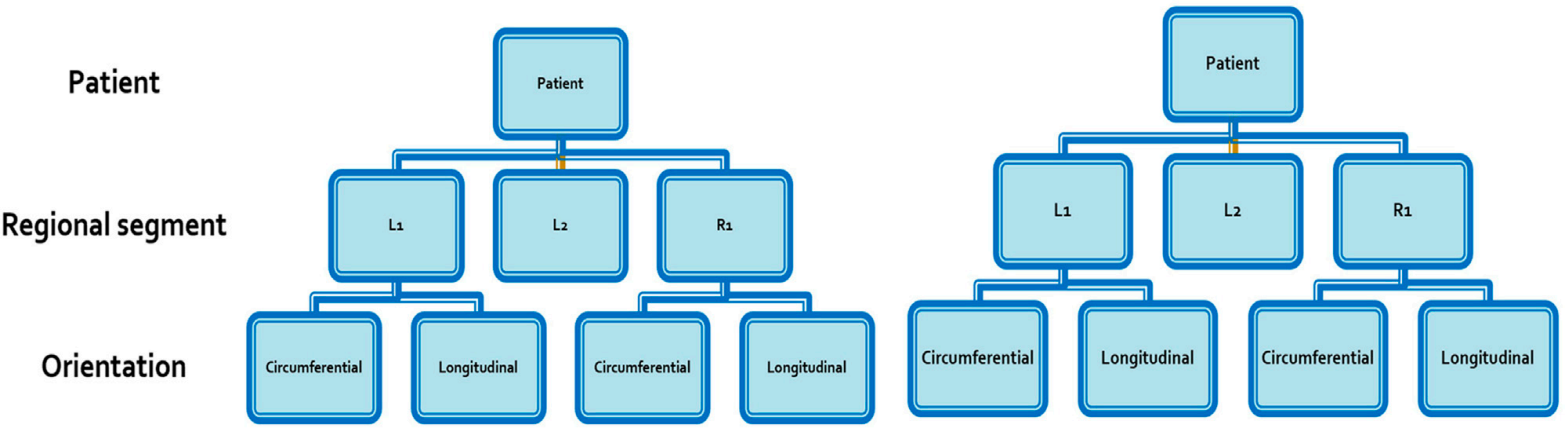
Hierarchical structure of data arising from each aortic segment.

As the aortic segments arose from among 10 different subjects, multilevel mixed-effect linear regression models were further constructed to account for the hierarchical structure of the data points. The data arising from each aortic segment was nested into a clustered hierarchical structure using two levels in the model: 1) patient; and 2) orientation of the subsection (circumferential versus longitudinal) (Figure 3). Univariate regression was again carried out, assessing the effect of WSS separately on wall thickness, UTS, MTS and histological parameters. The significance level for all models was set at *α* = 0.05.

## Results

Of the cohort of 10 patients, the majority were male (8/10), and of Caucasian ethnicity (8/10). Patient age was (mean ± standard deviation) 63.9years ±6.6 (Table 2). Whilst none had syndromic disease, 3 reported a family history of aortic disease. The mean aneurysm diameter was 54.7 mm ± 7.5. Three patients had root aneurysms, two patients had arch aneurysms, with the remaining patients having isolated ascending aorta aneurysms. All patients had good ventricular function (mean left ventricular ejection fraction 57.2% ± 9.0) with 3 patients suffering from severe aortic regurgitation. Maxima of jet velocity and area-averaged velocity at the model inlet were 1.86 ± 0.93 m/s and 0.51 ± 0.13 m/s (mean ± standard deviation), respectively.

**TABLE 2:** Summary of clinical covariates for recruited patients. AR = aortic regurgitation.

Covariate	Mean ± standard deviation	Covariate	N/10
Age (years)	63.9 ± 6.6	Female	2
		Peripheral vascular disease	2
Height (cm)	174.5 ± 12.5	Arch aneurysm	2
Weight (Kg)	84.7 ± 27.4	Root aneurysm	3
Body mass index (kg m^−2^)	27.2 ± 5.7	Severe AR	3
Body surface area (m^2^)	1.99 ± 0.27	—	—
		Smoking (current or ex-)	3
Left ventricular ejection fraction (%)	57.2 ± 9.0	Caucasian ethnicity	8
Mean arterial pressure (mmHg)	105.3 ± 19.3	Relevant family history	3
		Hypertension	4
Pulse wave velocity (m/s)	5.8 ± 0.7	Diabetes	0
Max aneurysm diameter (mm)	54.7 ± 7.5	Chronic airway disease	2

### WSS Distribution

**FIGURE 4:**
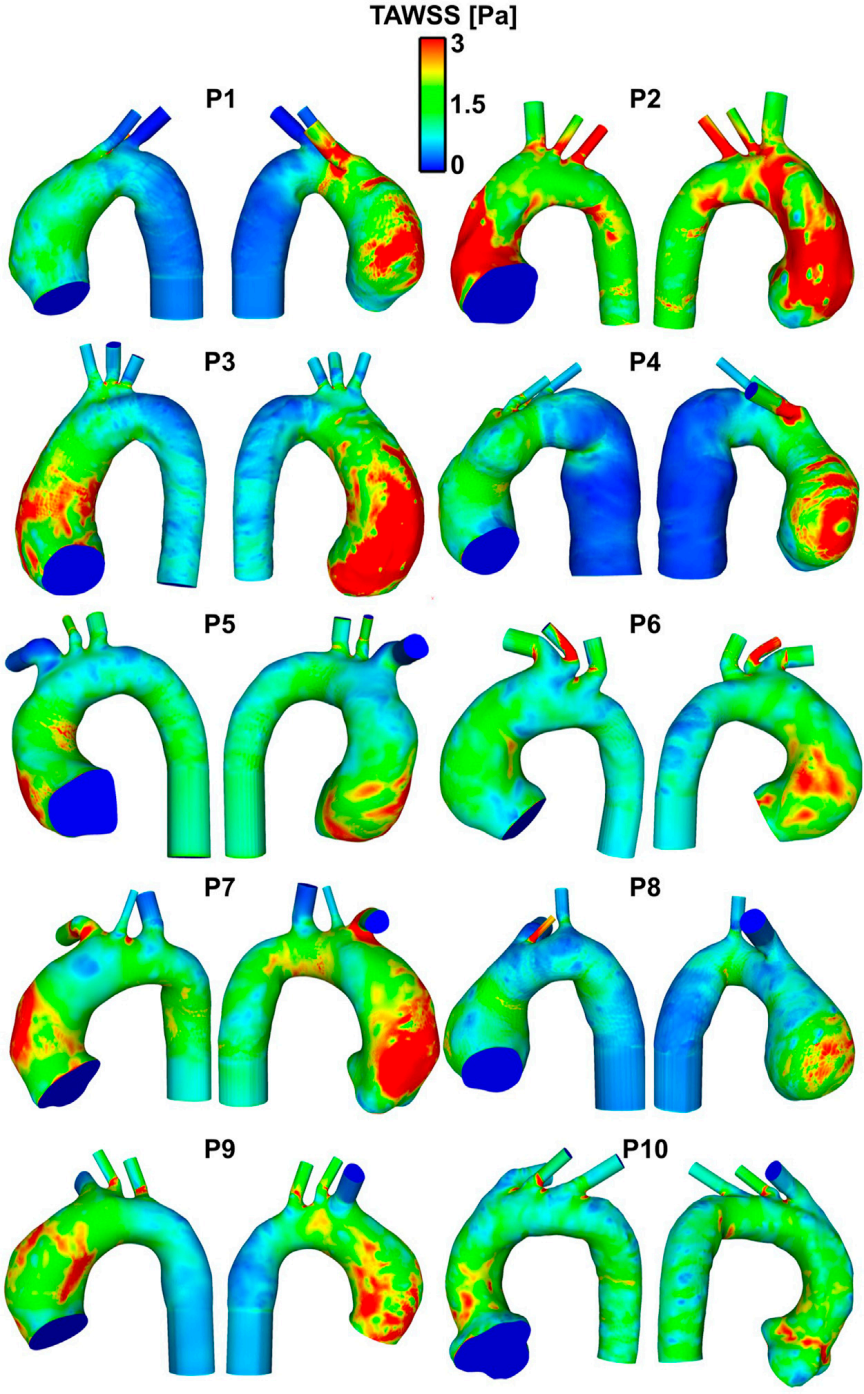
Patient-specific TAWSS distribution maps in the ten patients with ATAA. Regions in red indicate TAWSS >3 Pa, which, on gross inspection, are localized to the aneurysmal aorta, and particularly to the outer curve.

The patient-specific TAWSS distribution maps for all 10 patients are shown in Figure 4. The R1 segment (outer right wall) was the region yielding the highest values of WSS (Table 3), including temporal maximum WSS values (WSS_max_ and WSS_mean_) as well as time-averaged values (TAWSS_max_ and TAWSS_mean_). The highest value for WSS_max_ (24.98 ± 7.79 Pa) in the R1 region contrasted to the lowest value yielded from the inner curve (10.18 ± 4.14 Pa). When measuring the (peak in time) WSS averaged over space (WSS_mean_), the R1 region still yielded the highest value (11.68 ± 6.42 Pa). For TAWSS_max_, R1, also the region of highest WSS values, was 4.85Pa ± 2.07, compared to the inner curve (2.67 ± 0.81 Pa). This indicated that as well as the asymmetric peak flow patterns reached in systole acting on the potentially fragile intima in ATAA, the sustained stress over the cardiac cycle, as displayed by the TAWSS, was also asymmetrical and affecting the outer curve more intensely.

**TABLE 3:** Mean wall shear stress (WSS) parameters (±standard deviation) in anatomical regions around the aneurysm (averaged over 10 patients). All values are in Pascals (Pa).

	—	TAWSS _max_	TAWSS _mean_	WSS _max_	WSS _mean_
Lower	L1	4.15±1.30	2.29±0.99	22.31±5.05	8.49±4.17
	L2	3.12±1.37	1.71±0.74	16.03±6.66	5.69±3.33
	L3	3.06±1.28	1.83±0.87	13.95±8.08	6.22±4.12
Upper	L1	4.23±1.61	2.58±1.07	22.72±9.66	9.86±6.52
	L2	3.33±1.90	1.85±0.83	17.59±10.82	6.45±4.07
	L3	2.67±0.81	1.77±0.54	10.18±4.14	5.16±2.61
Lower	R1	4.85±2.07	2.60±1.03	24.98±7.79	10.11±4.90
	R2	4.63±2.21	2.23±0.87	23.73±11.47	8.51±5.04
	R3	3.04±1.07	1.56±0.38	14.51±7.54	4.27±1.69
Upper	R1	4.84±2.20	2.48±1.11	24.88±12.56	11.68±6.42
	R2	3.75±1.50	2.00±0.48	18.87±8.69	6.82±3.14
	R3	2.60±0.35	1.77±0.29	12.99±7.21	4.65±1.46

### Comparing WSS With Aortic Material Properties

**FIGURE 5:**
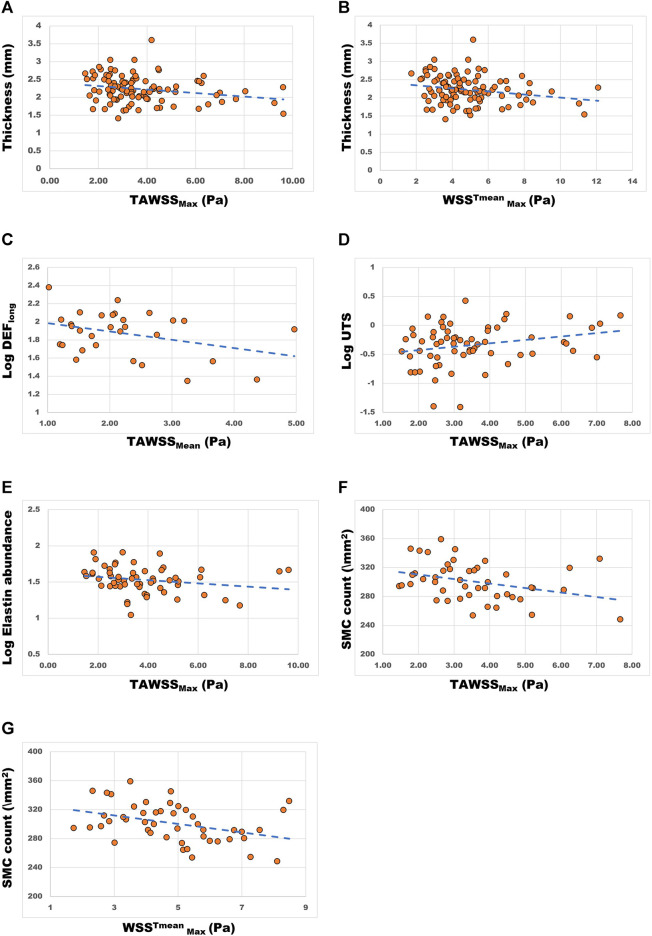
Scatter plots and trendlines demonstrating the relationship between WSS parameters (*x*-axes) and aortic wall material properties. **(A)** aortic wall thickness (mm) vs TAWSS_max_ (Pa) and **(B)** WSS^Tmean^_Max_ (Pa); **(C)** longitudinal dissection energy function (log_10_ transformed, Log DEF_long_) vs TAWSS_mean_ (Pa); **(D)** ultimate tensile strength (log_10_ transformed, Log UTS) vs TAWSS_max_ (Pa); **(E)** Elastin abundance (log_10_ (90-x) transformed) vs TAWSS_max_ (Pa); **(F)** Smooth muscle cell (SMC) count (\mm^2^) vs TAWSS_max_ (Pa) and **(G)** WSS^Tmean^_Max_ (Pa).

In order to test the hypothesis of flow mediated wall degeneration, WSS was compared with material properties of the aortic wall. Statistically significant linear regression analysis results are summarized in Table 4. Scatter plots and trendlines showing the relationship between WSS and aortic wall material properties are shown in Figure 5.

**TABLE 4:** Linear regression analysis: comparison of measurements of WSS per ATAA segment and tissue-derived parameters of corresponding segment from patient-specific excised tissue. circ = circumferential, long = longitudinal, SMC = smooth muscle cell. All results are statistically significant (*p*-value < 0.05).

Tissue measurement	WSS parameter	Coef	Standard error	95% CI	*p*-value
Tissue thickness	TAWSS_Max_	−0.0489	0.0209	−0.090–0.007	0.022
	WSS^Tmean^_Max_	−0.0421	0.0187	−0.079–0.005	0.026
Log dissection energy function (long)	TAWSS_Mean_	−0.211	0.106	−0.427–0.062	0.048
Log Ultimate tensile strength	TAWSS_Max_	0.136	0.067	0 0.001–0.270	0.048
Log Elastin abundance	TAWSS_Max_	−0.276	0.128	−0.531–0.020	0.035
SMC count	TAWSS_Max_	−6.19	2.59	−11.41–0.98	0.021
	${\text{WSS}}_{\text{Max}}^{\text{Tmean}}$	−5.87	2.11	−10.12–1.62	0.008

#### Wall Thickness

The data on measured wall thickness was normally distributed. Linear regression analysis was conducted which showed a statistically significant influence (*p* < 0.05) of TAWSS_max_ and WSS^Tmean^_Max_ parameters on wall thickness (Table 4). These yielded negative coefficients of variance, indicating that higher WSS values are associated with aortic wall thinning (Table 4; Figures 5A,B).

#### Peeling Properties

Peeling force (F_peel_) and dissection energy function (DEF) (as measured from tissue mechanical testing) provide a surrogate for the likelihood of dissection (i.e. separation of the medial layers) per region of interest. These were obtained from ATAA specimens in both the circumferential and longitudinal directions. Regression analysis (Table 4) comparing log transformed DEF values in the longitudinal orientation (DEF_long_) with TAWSS_Mean_ yielded a statistically significant relationship [coef −0.211, 95% CI (−0.427, −0.062), *p* = 0.048]. This relationship displayed a negative coefficient result: higher WSS values were associated with lower DEF_long_ values (as can also be observed in Figure 5C). In contrast, in the circumferential direction F_peel_ and DEF values showed an opposite trend, with higher values corresponding to higher WSS. However, results for F_peel_ (both longitudinal and circumferential) and circumferential DEF were not statistically significant.

#### Tensile Properties: Ultimate Tensile Strength (UTS) and Maximum Tangential Stiffness (MTS)

The comparison of log-transformed UTS data to TAWSS_max_ revealed a statistically significant positive correlation (coef 0.136, 95% CI [0 0.001, 0.270], *p* = 0.048) (Table 4; Figure 5D). A comparison of log-transformed MTS data to WSS revealed a nonsignificant trend toward higher values of WSS leading to stiffer aortic tissue (coef 0.120, 95% CI [-0.009, 0.248], *p* = 0.068). Comparisons to other WSS measures failed to show statistical significance. These results together suggest that high WSS leads to stiffer aortic tissue with higher tensile strength, but the statistical significance is marginal.

#### Microstructural Features

Elastin abundance was found to be lower in areas with higher TAWSS_Max_ [coef −0.276, 95% CI (−0.531, −0.020), *p* = 0.035] (Table 4; Figure 5E). An opposing, but statistically insignificant, trend was found for collagen abundance, i.e., higher TAWSS showed some association with areas of high collagen content. Higher WSS levels were also associated with lower counts of SMCs [TAWSS_Max_: coef −6.19, 95% CI (−11.41, −0.98), *p* = 0.021; WSS^Tmean^_Max_: coef −5.87, 95% CI (−10.12, −1.62), *p* = 0.008] (Table 4 and Figures 5F,G).

### Multi-Level Mixed Effects Regression

Significant results of multilevel mixed-effects linear models are reported in Tables 5, 6.

**TABLE 5:** Results of multilevel mixed-effects linear model for the main outcome of tissue thickness. The fixed effects part of the model tested the influence of TAWSS_max_ on thickness, whilst the random effect part of the model tested the influence of the patient. From these results, the influence of WSS alone on thickness does not occur in isolation and the variance occurring at the patient level is an important influencing factor.

	Coef	Standard error	95% CI	*p* Value
TAWSS_max_	−0.016	0.024	−0.063–0.031	0.496
Patient	0.032	0.019	0.010–0.105	—
Var (estimate tissue thickness)	0.100	0.015	0.074–0.133	—
Likelihood ratio test vs linear model	—	—	—	0.0002

**TABLE 6:** Multilevel mixed effects linear regression: association between wall shear stress (WSS^Tmean^_Max_) and smooth muscle cell (SMC) count. The fixed effects part of the model tests the effect of WSS^Tmean^_Max_ on SMC count. Random effects part of the model tests the effect of the data being nested with the patient domain.

	Coef	Standard error	95% CI	*p* Value
WSS^Tmean^_Max_	−4.87	2.240	−9.26–0.48	0.030
Patient	62.94	84.2	4.58–865.54	—
Var (estimate SMC count)	504.31	116.15	321.11–792.05	—
Likelihood ratio test vs linear model	—	—	—	0.167

#### Tissue Thickness

The fixed effects part of the model (i.e. the effect of TAWSS_Max_ on tissue thickness) was found to be non-significant (coef -0.016, 95% CI [-0.063, 0.031], *p* = 0.496) (Table 5). Thus, despite the linear relationship observed earlier between WSS and thickness, the data variance evident at the patient level remains significant.

#### Mechanical Properties: MTS and UTS.

In the case of UTS, the results trended towards a higher UTS in response to higher WSS, although this relationship was non-significant (*p* = 0.08). There was no evidence of influence of the patient or aortic tissue orientation on the WSS-UTS relationship. TAWSS_max_ was found to have no significant influence on MTS (*p* = 0.115).

#### Microstructural Features: SMC Count

TAWSS_max_ was found to have a persistently negative influence on SMC count [coef −4.87, 95% CI (−9.26, −0.48), *p* = 0.030]. The multi-level model found WSS^Tmean^_Max_ to be a stronger fit to the SMC count data than patient variance (Table 6). Multi-level mixed effects regression however found no significant association between the evaluated WSS parameters and elastin/collagen.

## Discussion

Computational modelling of thoracic aortic disease has expanded the repertoire of aneurysm diagnostics in recent years. Whilst several *in vivo* parameters related to aortic wall mechanics can be obtained from such models, their association with aortic wall mechanobiology, and in the pathogenesis of ATAA remains poorly understood. Further developments in this field may allow disease severity, progression, and acute events to be predicted at an individual patient level. This is required as isolated size measurements of the aorta are inadequate (Pape et al., 2007). Existing studies have associated abnormal WSS in the ATAA with aneurysm growth and wall degeneration (Geiger et al., 2012; Guzzardi et al., 2015; Condemi et al., 2017; Bollache et al., 2018). This study builds on such work, utilizing state of the art imaging, modelling, and biomechanical methods. However, because of the availability of pathologic human aortic specimens and the ability to assess their local properties, we made a more concerted effort to divide the aortic anatomy into finer areas for analysis, specifically including up to six regions circumferentially and a further upper and lower division (up to 12 in total).

### Elevated WSS on the Outer Curve

The WSS distribution in the ascending aorta is dominated by the curvature of the aortic arch, which forces blood emanating from the heart to change its direction. Fluid at the centre of the vessel is more difficult to displace as it is travelling at a higher velocity compared to that closer to the wall, so it is displaced to a greater degree (Caballero et al., 2013). Thus, blood is skewed towards the outer curvature of the bend.

Our findings agree with several previous studies assessing flow in ATAA, which also noted the highest WSS on the outer curve (Bieging et al., 2011; Bürk et al., 2012; Van Ooij et al., 2015).

### WSS and Wall Thickness

Owing to the low temporo-spatial resolution of cross-sectional imaging and difficulty in tracking the motion of the aortic wall (<2.5 mm thickness), estimations of stress distribution within the aneurysm wall are reliant on important assumptions of aortic wall thickness (commonly assumed to be constant) and material properties (commonly not patient- or even disease-specific) (Martin et al., 2015; Gomez et al., 2020). Our results have identified a potential link between areas of high WSS and aortic wall thinning. Reduced wall thickness in areas of high WSS has been previously shown in femoral artery bifurcation by Kornet et al. (1999), who explained this finding with the link between low WSS, increased influx of substances into the aortic wall (through an increase in blood residence time) and increased release of vasoactive molecules potentially causing thickness increase of the intima-media layer.

These results represents a step towards the incorporation of wall thickness inferences from baseline imaging that can be incorporated into fluid-structure interaction models and estimations of wall stress distribution. These results also help to strengthen the flow-mediated degeneration hypothesis, whereby persistently elevated WSS throughout the cardiac cycle exposes mechanocytes within the aortic wall to prolonged stimuli and downstream potentially maladaptive remodeling (Humphrey et al., 2015). However, the conducted hierarchical analysis also suggests that data variance at the patient level remains significant.

### WSS and Aortic Wall Mechanical Properties

Our analyses on the effect of WSS on wall mechanical properties produced two main findings. Firstly, higher WSS was associated with a reduced DEF in the longitudinal direction. This contrasted with circumferential DEF, which showed an insignificant increasing trend. This further exemplifies the anisotropic nature of the aortic wall and suggests that it extends to influence both tear direction and location in aneurysm dissection (Manopoulos et al., 2018). Secondly, the aortic wall had significantly higher values for UTS with higher WSS, indicating a reduced likelihood for wall rupture in response to elevated WSS. In addition, the aorta might be stiffer in response to high WSS, as suggested by elevated values for MTS. This result, however, was not statistically significant.

### WSS and Microstructural Features: Implications for Mechanobiology

Higher WSS levels were found to be associated with a reduction in both elastin abundance and smooth muscle cell (SMC) count. Loss of elastin integrity and relative increase in collagen describes the reduced compliance and increased stiffness of the aorta seen in ageing (Wagenseil et al., 2009). Studies have shown that global increases in vascular structural stiffness reflect increased central pulse pressures and pulse wave velocities (Lacolley et al., 2009; Safar et al., 2010) that pathologically increase proximal aortic loading. Collagen deposition in many cases of ATAA has been observed to be higher, reflecting the compensatory fibrotic changes as a result of the disease process (Wågsäter et al., 2013; Meng et al., 2014). The microstructural response to elevated WSS therefore explains these changes (Humphrey et al., 2015), and describes the resulting aortic wall stiffness and delamination potential, thus increasing the likelihood of dissection (Phillippi et al., 2011).

In addition to endothelial cells and fibroblasts, SMCs are part of the repertoire of crucial mechano-sensing and -regulating cells in the aortic wall. These cells display adaptive remodeling in response to shear stress encountered by endothelial cells, which they detect with integrins, glyclocalyx, membrane microdomains, cytoskeleton, receptor tyrosine kinases and others. The direct link between WSS level and SMC found in the present study is likely to result from mechanodysregulation in response to elevated WSS, involving a disruption of cell matrix connections which are vital to aortic wall integrity (Michel et al., 2018). Dysfunctional mechanosensing can lead to cellular apoptosis and/or an atrophic remodeling response, thus disturbing the structural integrity of the aortic wall (Leung et al., 1976). Whilst there are a number of possible intracellular and matrix signaling pathways associated with the process that we have not tested for, they are likely to culminate in a final common pathway, leading to cellular loss and matrix degeneration.

### Strengths and Limitations

This study benefits from a robust method of ATAA flow-to-tissue patient-specific association, which arises from segmental aneurysm analysis. Conducting the tissue characterization and CFD portions of work separately has helped reduce the risk of bias, that could result from basing aortic tissue acquisition on findings from flow analysis retrospectively (Guzzardi et al., 2015). Our statistical methods have aimed to appreciate the spread of data and made use of multilevel regression models, which have not been utilized in similar studies. Altogether this aims to improve the validity of the findings.

The study is limited by its small sample size. This may perhaps explain the lack of significance in some relationships. Larger studies would also help deal with potential confounders such patient covariates and valve function. In addition, CFD simulations were conducted under rigid wall assumption (i.e. aortic wall compliance was not taken into account). Including aortic wall compliance could further improve WSS estimation. However, this would significantly increase the computational time required to conduct patient-specific simulation further decreasing the likelihood of adoption of this technique into the clinic. Rigid wall is therefore a common assumption is several computational studies (Morbiducci et al., 2013) with a translational goal. In addition, we do not expect WSS results to be significantly affected by this assumption. Firstly, because our mechanical test results suggest increased stiffness in regions of enhanced WSS. Secondly, previous imaging studies have shown a reduction in aneurysm wall compliance when compared with healthy tissues. Larger sample sizes and the incorporation of computational methods to couple flow and wall material properties will form the basis of future work.

## Conclusion

Our findings make a strong case for the co-localization of elevated WSS and patterns of medial degeneration, the hallmark of TAA disease. This is likely a result of the negative remodeling process of the aortic wall in response to chronic exposure to locally higher shear forces. Detailed wall shear stress analysis could predict areas of altered vascular wall mechanics and microstructural features in ascending aortic aneurysms. Presented findings further validate 4D-flow MRI and computational fluid dynamics as powerful tools for risk stratification in aneurysmal disease. This can improve precision in the timing and planning of intervention in at-risk patients.

## Data Availability

The raw data supporting the conclusions of this article will be made available by the authors, without undue reservation.

## Supplementary Material

Supplementary Figure 1S Example of unstructured mesh used for geometry discretization. The image shows details of the tetrahedral core and 10 prismatic layers at the wall **(A)** and of local mesh refinements. These were prescribed at the arch branches and the aneurysm wall, where a finer mesh was designed where higher velocity gradients were observed from 4D flow MRI data. Smooth transition between finer and coarser regions was ensured by prescribing small growth ratios and verified through mesh expansion factor (Ansys ICEM) **(B)**.
