# Supplementary figures and images for: High Wall Shear Stress can Predict Wall Degradation in Ascending Aortic Aneurysms: An Integrated Biomechanics Study
Source: Front Bioeng Biotechnol. 2021 Oct 18;9:750656. doi: 10.3389/fbioe.2021.750656 (PMC8558434; doi:10.3389/fbioe.2021.750656)

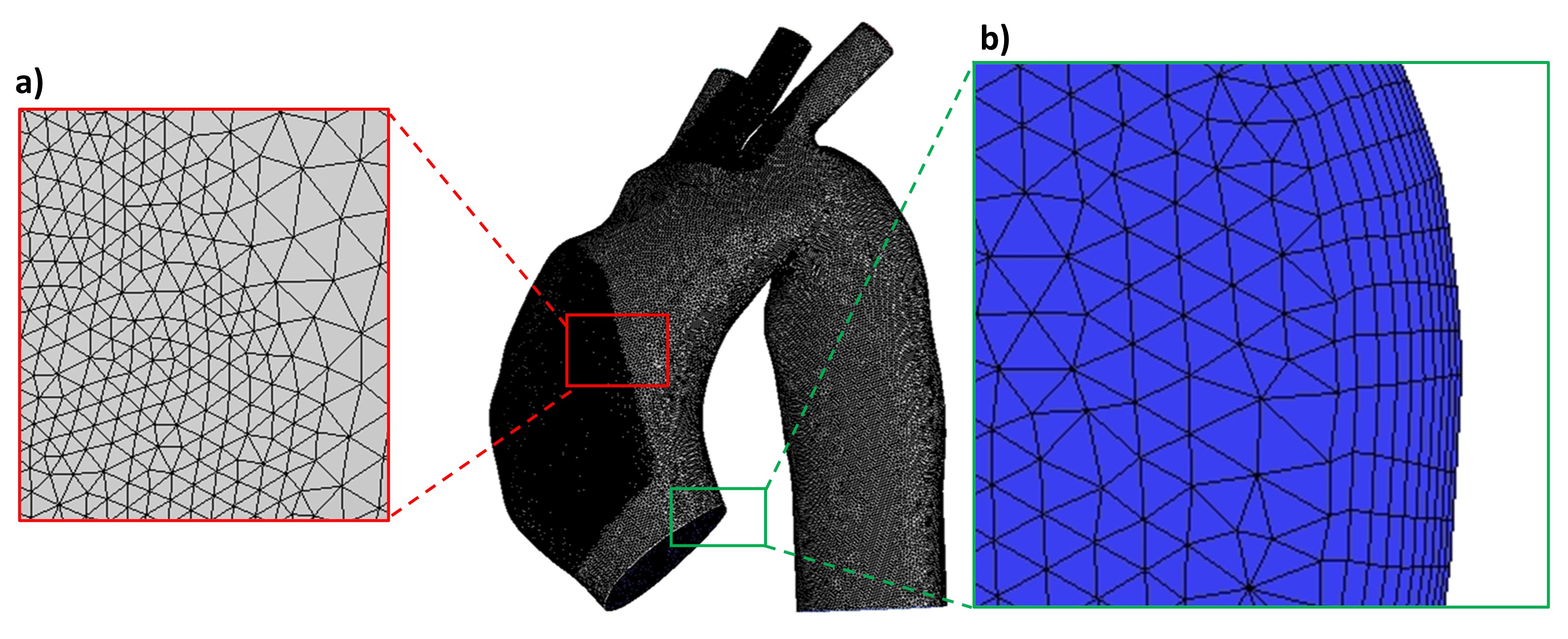

Supplement: Supplementary file 1 [file Image1.JPEG]
